# Kisspeptin/kisspeptin receptor system in pseudopregnant rabbit corpora lutea: presence and function

**DOI:** 10.1038/s41598-019-41623-1

**Published:** 2019-03-25

**Authors:** Margherita Maranesi, Linda Petrucci, Leonardo Leonardi, Antonello Bufalari, Francesco Parillo, Cristiano Boiti, Massimo Zerani

**Affiliations:** 1 Dipartimento di Medicina veterinaria, Università di Perugia, via San Costanzo 4, Perugia, IT 06126 Italy; 2 Scuola di Bioscienze e Medicina veterinaria, Università di Camerino, via Circonvallazione 93, Matelica, IT 62024 Italy

## Abstract

Kisspeptin (KiSS) and its related receptors (KiSS1R) have a critical role in the reproduction of mammals. The KiSS/KiSS1R system is expressed in numerous reproductive organs including the ovary. Here, we studied the expression of the KiSS/KiSS1R system and its functional role in rabbit corpora lutea (CL) at days 4 (early-), 9 (mid-), and 13 (late-stage) of pseudopregnancy. *In vitro* progesterone, prostaglandin (PG) F2α (PGF2α) and E2 (PGE2) productions and prostaglandin-endoperoxide synthase 1 (PTGS1) and 2 (PTGS2) activities were evaluated. Immune reactivity (IR) for KiSS and KiSS1R were detected in luteal cells at nuclear and cytoplasmic level at all luteal stage for KiSS and only at early- and mid-stage for KiSS1R; IR decreased from early- to later stages of pseudopregnancy. The KiSS-10 augmented progesterone and PGE2 and diminished PGF2α secretions by early- and mid-CL; KiSS-10 reduced PTGS2 activity at early- and mid-stages, but did not affect PTGS1 at any luteal stages. The antagonist KiSS-234 counteracted all KiSS-10 effects. This study shows that the KiSS/KiSS1R system is expressed in CL of pseudopregnant rabbits and exerts a luteotropic action by down-regulating PTGS2, which decreases PGF2α and increases PGE2 and progesterone.

## Introduction

The hypothalamic neuropeptides kisspeptins (KiSS) and its cognate receptors (KiSS1R) have a central role in the reproduction of mammals through the regulation of gonadotropin-releasing hormone (GnRH) production^1^ necessary to attain puberty and maintain normal reproductive function^2^.

The *KiSS* gene codes a precursor peptide that is cleaved to a 54 amino acid peptide, which can be processed into shorter fragments consisting of 14, 13, and 10 amino acids (KiSS-14, KiSS-13, and KiSS-10, respectively)^3,4^. These three peptides, which share a similar conserved region at the C-terminal sequence of Arg-Phe-NH2, are currently indicated as KiSS^5^. Kisspeptins bind the G-protein coupled receptor 54 (currently known as KiSS1R)^6^, whose expression was first observed in the hypothalamus of rats and humans^4^.

Increasing evidences indicate that KiSS and KiSS1R are expressed at gene and protein levels in the ovary and corpora lutea (CL) of several mammalian species^7–9^. Following binding to its cognate receptor, KiSS can be involved in the regulation of CL lifespan by acting on key steroidogenic enzymes that modulate progesterone production^9^. More recently, Laoharatchatathanin *et al*.^10^ confirmed this hypothesis suggesting an involvement of KiSS also in the luteinization of rat granulosa cells.

The rabbit is an excellent animal model to investigate the mechanisms regulating the function of CL because ovulation, and consequently pseudopregnancy, can be timed precisely with the injection of GnRH^11^. Thus, the main objectives of this research were (i) to verify the modulatory effects, if any, of KiSS on rabbit luteal function at different stages of pseudopregnancy and, eventually, (ii) understand the underlying physiological mechanisms. Consequently, we investigated the expression of KiSS and KiSS1R in CL obtained at days 4, 9, and 13 of pseudopregnancy, together with the effects induced by KiSS in CL cultured *in vitro* on progesterone, prostaglandin (PG) E2 (PGE2), and F2α (PGF2α) production and on the activity of prostaglandin-endoperoxide synthase 1 and 2 (PTGS1 and PTGS2, respectively).

## Results

### KiSS1 and KiSS1R expression

**Figure 1:**
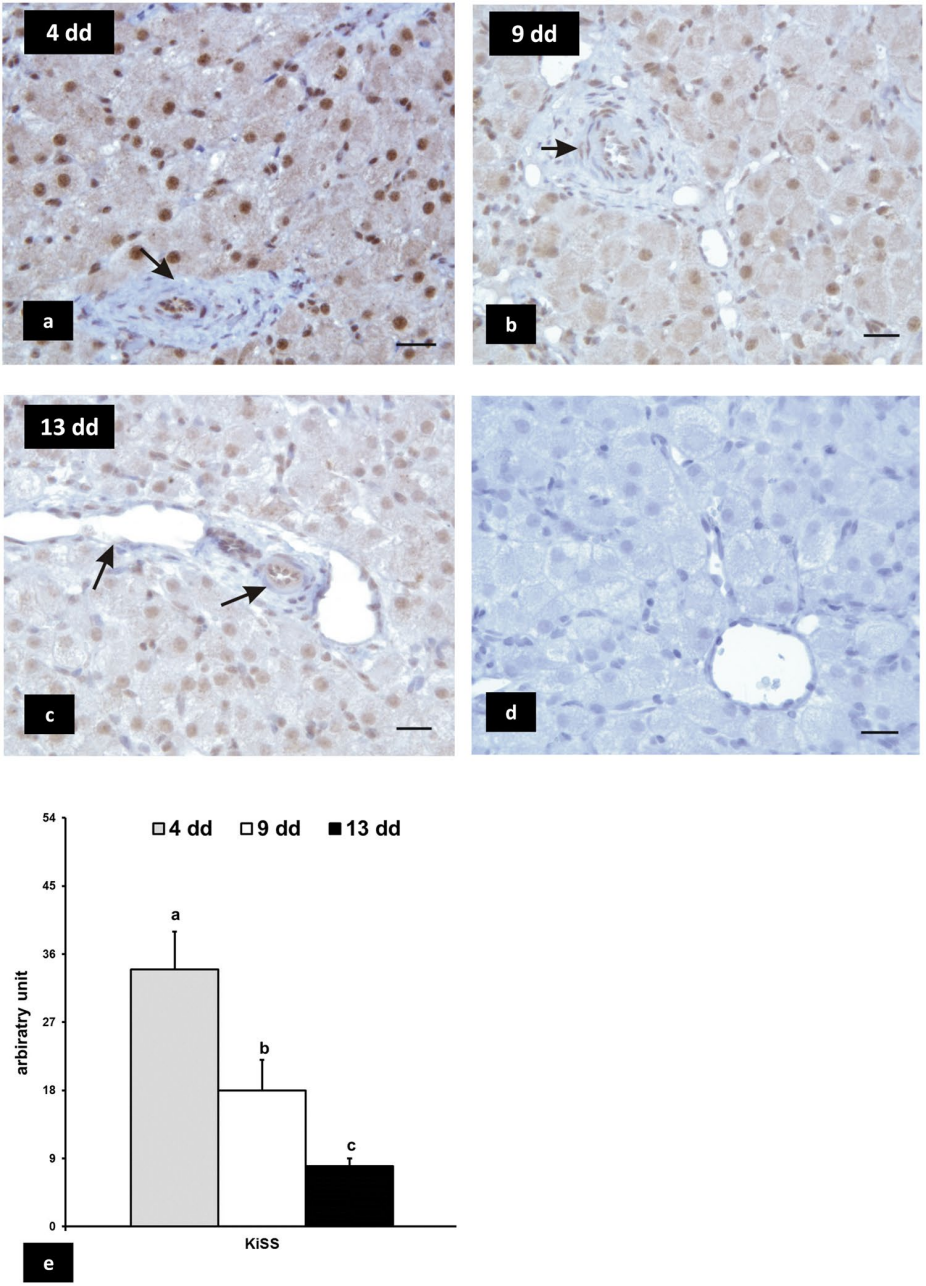
Rabbit CL, immunoreactivity of KiSS. The immunostaining is intensely localized in the nucleus and cytoplasm of luteal cells at early stage (4 days) of pseudopregnancy (panel a), it is moderate at mid stage (9 days) (panel b) and weak at late stage (13 days) (panel c). Endothelial and blood vessel smooth muscular cells are immunopositive (arrows). The immunopositivity is abolished in negative control (panel d). Bars = 20 μm. Panel e: densitometric analysis of immunostaining intensity. Values are the means ± SD of thirty replicates (one-way ANOVA). Different letters above the bars indicate significantly different values (p < 0·01).

**Figure 2:**
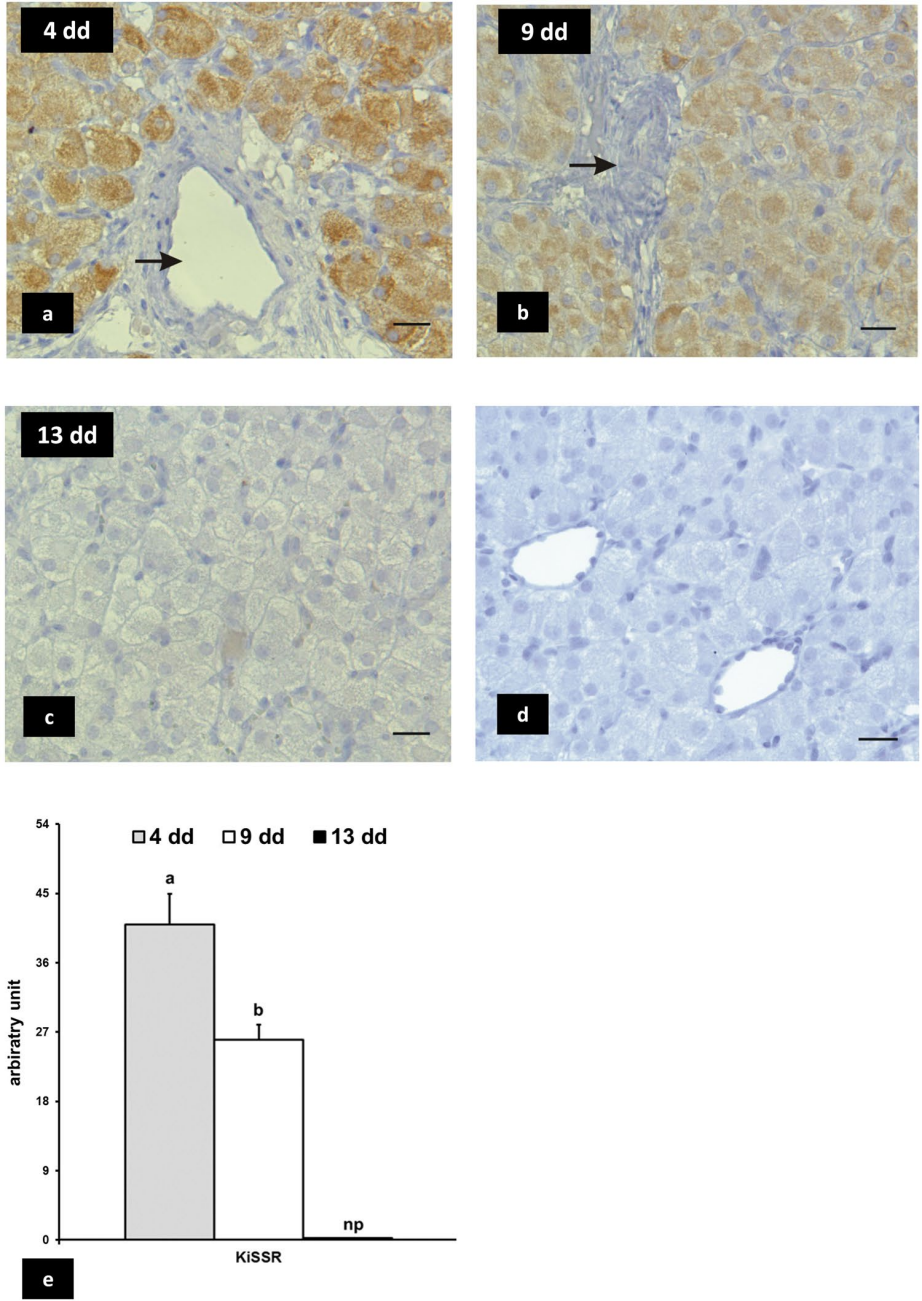
Rabbit CL, immunoreactivity of KISS1R. The immunosignals are detected intensely in the cytoplasm of luteal cells at early stage (4 days) of pseudopregnancy (panel a) and moderately at mid stage (9 days) (panel b); luteal cells are immunegative at late stage (13 days) (panel c). Blood vessels are not immunostained (arrows). The immunopositivity is abolished in negative control (panel d). Bars = 20 μm. Panel e: densitometric analysis of immunostaining intensity. Values are the means ± SD of thirty replicates (one-way ANOVA). Different letters above the bars indicate significantly different values (p < 0·01).

KiSS1 immunoreactivity was revealed in the nucleus and cytoplasm of luteal, endothelial, and smooth muscular cells of blood vessel at all stages of pseudopregnancy (Fig. 1, panels a,b,c). The number of KiSS1 positive cells declined (p < 0.01) progressively with the ageing of CL (Fig. 1, panel e). Positive immune-like signals for KiSS1R were identified in the cytoplasm of luteal cells at early- and mid-stages of pseudopregnancy (Fig. 2, panels a,b), whereas they were not revealed at late-luteal stage (Fig. 2, panel c). The number of positive cells was higher (p < 0.01) in early stage CL (Fig. 2, panel e). The wall of blood vessels did not immunoreact with KiSS1R at all stages of pseudopregnancy (Fig. 2 panels a,b,c).

No staining was evidenced in CL sections when the primary antibodies were not added or replaced with mouse or goat IgG (negative controls: Fig. 1, panel d; Fig. 2, panel d).

The presence of positive immunosignals for KiSS and KiSS1R in hypothalamic neurons localized near the III ventricle validated the specificity of KiSS and KiSS1R antisera utilised in the immunohistochemical study (Fig. S1).

### Western Blotting

**Figure 3:**
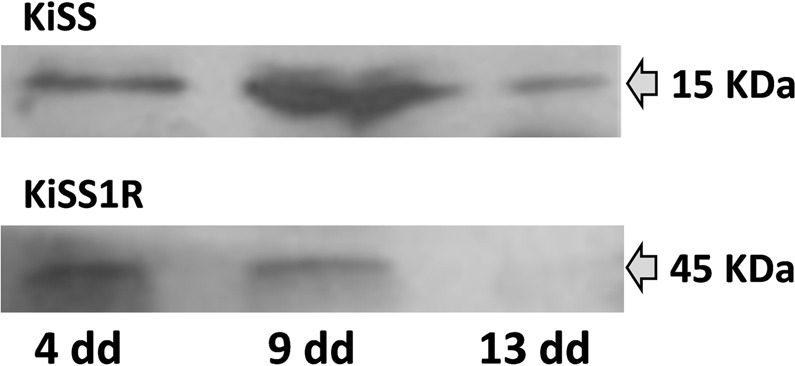
Representative different immunoblots of KiSS (upper panel) and KiSS1R (lower panel) in CL lysates at different luteal stages (4, 9, and 13 days of pseudopregnancy).

Western blotting confirmed the specificity of antisera used for the KiSS and KiSS1R immunohistochemistry, showing in all luteal stages examined typical bands at about 15 kDa and 45 kDa, respectively, except for late CL that did not show the latter band (Fig. 3).

### Hormone Production *in Vitro*

**Figure 4:**
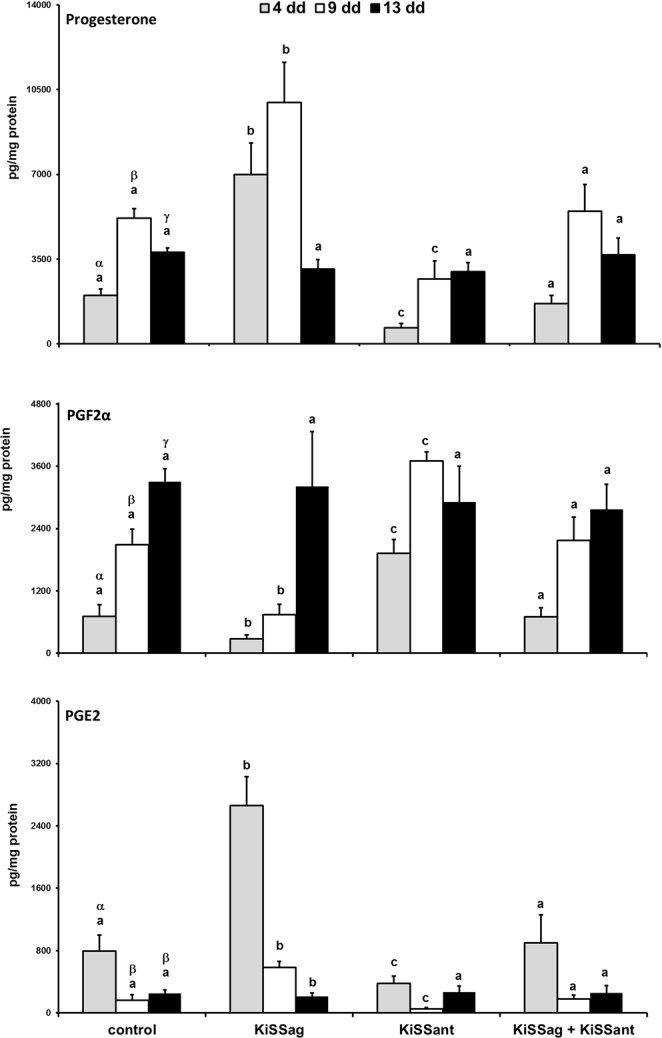
*In vitro* effects of KiSS agonist (KiSS-10, KiSSag) and antagonist (KiSS-234, KiSSant) on progesterone (upper panel), PGF2α (middle panel), and PGE2 (lower panel) release by rabbit CL at days 4, 9, and 13 of pseudopregnancy. Values are the means ± SD of five replicates (two-way ANOVA). Different letters above the bars indicate significantly different values (p < 0·01): Greek letters among control CL days, Latin letters among experimental groups of the same CL day.

In the control group, early- and mid-stage CL released into the medium the lowest (p < 0.01) and the highest (p < 0.01) amount of progesterone, respectively (Fig. 4, upper panel). Addition of KiSS-10 into incubation wells increased (p < 0.01) progesterone secretion by early- and mid-stage CL, while addition of KiSS-234 had an opposite (p < 0.01) effect (Fig. 4, upper panel).

Basal PGF2α secretion rose (p < 0.01) with the ageing of CL, being lower in early-, intermediate in mid-, and higher in late-stage of pseudopregnancy (Fig. 4, mid panel). Addition of KiSS-10 into incubation wells decreased (p < 0.01) PGF2α secretion by early- and mid-stage CL, while that of KiSS-234 enhanced (p < 0.01) its production (Fig. 4, mid panel).

Basal PGE2 production was greater in early-stage CL (p < 0.01) than in mid- and late-ones (Fig. 4, lower panel). Addition of KiSS-10 agonist augmented (p < 0.01), while that of the KiSS-234 antagonist decreased (p < 0.01) PGE2 secretion by early- and mid-stage CL (Fig. 4, lower panel).

KiSS-10 plus KiSS-234 did not affect progesterone, PGF2α, and PGE2 basal productions (Fig. 4).

### Luteal Enzyme Activities

**Figure 5:**
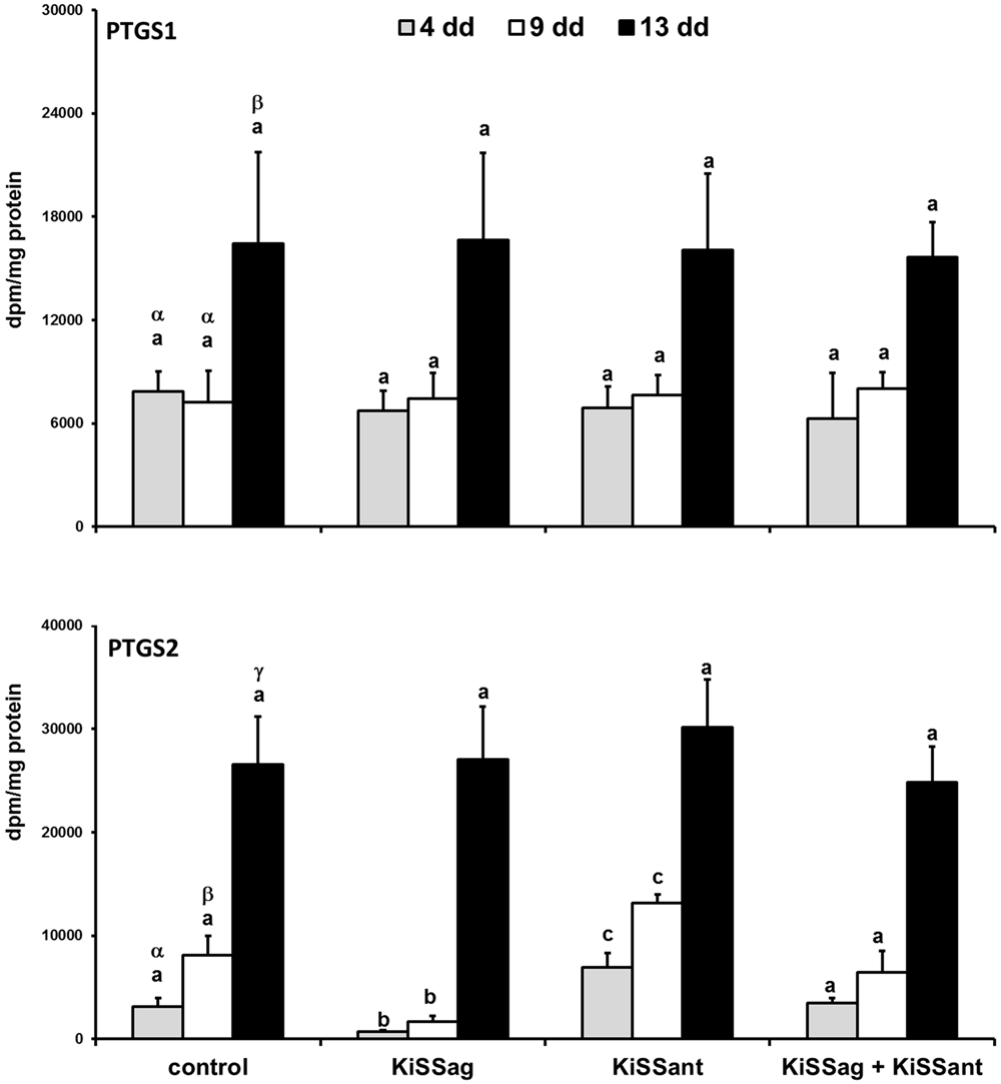
*In vitro* effects of KiSS agonist (KiSS-10, KiSSag) and antagonist (KiSS 234, KiSSant) on PTGS1 (upper panel) and PTGS2 (lower panel) activities by rabbit CL at days 4, 9, and 13 of pseudopregnancy. Values are the means ± SD of five replicates (two-way ANOVA). Different letters above the bars indicate significantly different values (p < 0·01): Greek letters among control CL days, Latin letters among experimental groups of the same CL day.

The activity of PTGS1 was greater (p < 0.01) in late-stage CL than in early- and mid-ones (Fig. 5, upper panel). The basal activity of PTGS2 increased (p < 0.01) with the advancement of luteal stages (Fig. 5, lower panel). The KiSS-10, KiSS-234, and KiSS-10 plus KiSS-234 did not modify the PTGS1 activity independently of luteal stages (Fig. 5, upper panel). The KiSS-10 reduced (p < 0.01) and KiSS-234 rose (p < 0.01) the luteal activity of PTGS2 at early- and mid-luteal stages (Fig. 5, lower panel), whereas KiSS-10 plus KiSS-234 did not affect this enzyme activity in any CL, independently of luteal stage (Fig. 5).

## Methods

### Reagents

Mouse monoclonal primary antibody anti-KiSS and goat polyclonal anti-KiSS1R were acquired from AbD Serotec (cat. n. MCA3219Z, Kidlington, UK) and from Santa Cruz Biotechnology (cat. n. sc-48219, Santa Cruz, CA, USA), respectively. Biotin goat anti-mouse IgG and biotin horse anti-goat IgG secondary antibodies, avidin-biotin complex (ABC), chromogen 3,3′-diaminobenzidine tetrachloride (DAB), and mouse- and goat-IgG were purchased from Vector Laboratories (Burlingame, CA, USA). Chromogen 3-amino-9-ethylcarbazole (AEC) was obtained by Dako Products (Santa Clara, CA, USA). The horseradish peroxidase (HRP)-conjugated IgG secondary antibodies used for western blot (WB) were purchased from Thermo Fisher Scientific (Rockford, IL, USA). Protran nitrocellulose membranes were acquired from Whatman (Dassel, Germany). Biomax films used to document immunocomplexes were obtained from Kodak Laboratories (Rochester, NY, USA). The enhanced chemiluminescence detection system for WB (Immobilon Western Chemiluminescent HRP Substrate) was acquired from Millipore (Billerica, MA, USA). The protein assay kit was purchased from Bio-Rad Laboratories (Hercules, CA, USA) and incubation wells from Becton Dickinson Co. (Clifton, NJ, USA). Medium 199 and Earles Balanced Salt Solution were acquired from GIBCO (Grand Island, NY, USA), whereas HEPES and BSA were from Sigma. Tritiated hormones and [^3^H]AA were obtained from Amersham Biosciences (Amersham Biosciences Ltd, Little Chalfont, Bucks, UK), while progesterone, PGF2α, and PGE2 antisera, and non-radioactive hormones came from Sigma (St Louis, MO, USA). Selective PTGS2 (NS-398) and non-selective PTGS inhibitors (acetylsalicylic acid) were acquired from Sigma-Aldrich. All other chemicals were obtained from local companies.

### Animals and hormone treatments

For this study, sexually mature unmated New Zealand White female rabbits (3.5–4 kg BW, aged 5 months) were used. The does were housed at the experimental farm of the University of Perugia in individual metal cages under a constant photoperiod of light (14 L:10D; lights off at 21.00 h) and a temperature of 18 to 24 °C. Rabbits were fed *ad libitum* a commercial diet and water was provided *ad libitum*. Pseudopregnancy was induced by an IM injection of eCG (20 IU; Folligon, Intervet, Holland) followed 3 days later by an IM injection of GnRH analogue (buserelin 0.8 µg, Receptal, MSD Animal Health, Italy)^11^. The day of GnRH administration was imposed as day 0. At day 4 (early-), 9 (mid-), and 13 (late-stage) of pseudopregnancy, 6 rabbits for each stage were killed by cervical dislocation.

### CL collection and processing

Few minutes after sacrifice, CL were harvested from ovaries (~12 CL/ovary), repeatedly washed with saline and carefully cleaned from non-luteal tissue; thereafter, CL were either promptly processed for *in vitro* experiments or rinsed with PBS and frozen at −80 °C for Western blotting analysis. The ovaries of two rabbits for each luteal stage were fixed in 4% (w/v) formaldehyde in PBS (pH 7.4) for 24 h at room temperature and then processed for the immunohistochemical study^12^. The hypothalamic tissue samples were obtained from three rabbits to evaluate the specificity of antisera.

### Immunohistochemistry of KiSS and KiSS1R

The samples were fixed for 24 h and then dehydrated with graded ethanol according to routine histological techniques, embedded in paraffin wax and sectioned serially at a thickness of 4 μm. Once deparaffinised, rehydrated via graded ethanol, and rinsed in distilled water, the sections were microwaved in citrate buffer (10 mM, pH 6) for antigen retrieval^13^. The sections were then rinsed with Tris-buffered saline (TBS), dipped for 1 h in 3% H_2_O_2_ methanol and rinsed again in TBS. To prevent background labelling, the sections were incubated for 30 min at room temperature with normal goat serum (1:10) for anti-KiSS and normal horse serum (1:10) for anti-KISS1R. Thereafter, the sections were incubated overnight at 4 °C in a moist chamber with primary antibody: mouse monoclonal anti-KiSS (1:100) and goat polyclonal anti-KiSS1R (1:50) diluted in TBS with Triton X-100 (0.2%) and bovine serum albumin (BSA, 0.1%). The next day, after three TBS washes, the slides were exposed again to normal serum and then to biotin goat anti-mouse or to biotin horse anti-goat IgG conjugate (1:200) for 30 min at room temperature for KiSS and KiSS1R, respectively. Following TBS rinses, the sections of ovarian tissue were treated for 30 min with avidin-biotin complex (ABC kit), whereas those of hypothalamic tissue with AEC plus substrate-chromogen. After TBS rinsing, the active peroxidase sites were visualized using the DAB kit as chromogen. The sections were washed twice for 5 min with distilled water and, in some cases, counterstained with Mayer’s haematoxylin, and mounted for light microscopy following standard protocols^12^.

Hypothalamic tissue sections, processed as above, were used as positive control of specific staining. Tissue sections where the primary antibody was absent or replaced with mouse or goat IgG were used as negative controls of non-specific staining. The intensity of immune reactions was evaluated with an image analyser (IAAS 2000, Delta Sistemi, Rome, Italy) as described in a previous work^14^.

### Western Blot of KiSS and KiSS1R

The expression of KiSS and KiSS1R proteins were analysed by WB in luteal tissue of early-, mid-, and late-stages. Proteins were extracted from pooled CL homogenized in 1 mL of ice-cold RIPA buffer as previously described^15^.

After incubation (4 °C, 20 min), the homogenate samples were centrifuged at 12,000 × *g* for 60 min at 4 °C. The concentration of protein in each supernatant was assayed with the protein kit. Same amounts of protein (20 μg) were separated by discontinuous 10% SDS-PAGE gel electrophoresis (4% staking gel, 40 min at 200 V and 500 mA).

Proteins were then transferred onto nitrocellulose membranes for 1 h at 100 V and 350 mA. Next, membranes were blocked in TBS with Tween-20, non-fat dried milk, and BSA (0.05, 5, and 3%, respectively). The transferred proteins were probed with anti-KiSS and anti-KiSS1R antibodies (1:500) overnight at 4 °C and thereafter with HRP-labelled secondary antibody (1:20,000) for 1 h at room temperature under gentle agitation. Antibody incubations were done in TBS as above. The immune blotting substrates were detected by enhanced chemiluminescence according to the manufacturer’s instructions and exposed to X-ray film. Blot images were scanned and acquired.

### *In Vitro* Experiments

The *in vitro* experiment was performed as previously reported^16^. Briefly, early-, mid-, or late-CL were randomly distributed (one CL/well) into incubation wells containing 1 ml of culture medium 199 with Earles Balanced Salt Solution, 2.2 mg/ml sodium bicarbonate, 2.3 mg/ml HEPES, and 3% BSA (w/v). Before treatment, CL were quartered inside each well using fine forceps. For each experimental set, there were 4 treatment groups (5 wells/group) as follows: (I) medium alone as control, (II) KiSS agonist (KiSS-10, 200 nM), (III) KiSS antagonist (KiSS-234, 50 nM), and (IV) KiSS agonist (200 nM) plus KiSS antagonist (50 nM). After incubation (4 h at 37 °C in air with 5% CO_2_), the media of each well were collected and frozen at −20 °C for later determination of progesterone, PGF2α, and PGE2 concentrations. The CL were weighed and their protein concentrations determined using the protein assay kit according to manufacturer’s instructions. Preliminary experiments defined the optimal condition for incubation and the effective doses for KiSS agonist and antagonist here used (Fig. S2).

### Hormone Assays

Progesterone, PGF2α, and PGE2 concentrations in culture media were assayed by RIA as described in a previous work^17^. Intra- and inter-assay coefficients of variation and minimum detectable doses were: 6.1%, 8.9%, and 11 pg for progesterone; 7.5%, 12.4%, and 18 pg for PGF2α and 6.4%, 12.2%, and 16 pg/ml for PGE2.

### Determination of Enzyme Activity

The enzymatic activity of PTGS1 and PTGS2 was evaluated by quantifying the rate of the [^3^H]AA disappearance as reported in a previous work^14^. The activity of PTGS1 in each CL was determined by establishing the disappearance values of [^3^H]AA following incubation with NS-398 (1 μm), a selective inhibitor of PTGS2. Conversely, the corresponding activity of PTGS2 was derived by computing the rate of [^3^H]AA loss following incubation without selective PTGS2 inhibitor (acetylsalicylic acid, 1 mM), and subtraction of the PTGS1 value. The activities of PTGS1 and PTGS2 were adjusted by detracting basal [^3^H]AA loss rates caused by non-specific enzymatic reactions.

### Statistical Analysis

Data were analysed by one-way or two-way ANOVA (treatments *vs*. luteal stages, Table 1) and multiple comparisons were performed with Student-Newman-Keuls post hoc t-test. Differences with a probability level of p < 0.01 were considered to be statistically significant.

**Table 1:** Two-way ANOVA *F* values: treatments (control, KiSS agonist, KiSS antagonist, KiSS agonist + KiSS antagonist) *vs*. CL stages (early, mid, late).

	Treatments	CL stages	Interaction
Progesterone	89.9*	82.6*	25.7*
PGF2α	26.8*	116.8*	12.0*
PGE2	85.2*	220.0*	49.7*
PTGS1	0.1	66.1*	0.2
PTGS2	17.0*	417.8*	2.6

DF: treatments, 3; CL stages, 2; interaction, 6; error, 48, *p < 0.01.

### Ethics approval and consent to participate

The protocols involving the use of the animals for these experiments were approved by the Bioethic Committee of the University of Perugia and were performed in compliance with Italian law (DL 116/92) and EEC directive on animal research (N. 86/609/EEC).

## Discussion

To our knowledge, this is the first study to show that the KiSS/KiSS1R system may control the lifespan of rabbit CL by modulating differently progesterone and prostaglandins synthesis and PTGS2 activity during pseudopregnancy.

Besides the central nervous system, many tissues express both KiSS and KiSS1R, including the ovary^2,18^. The wide distribution of the KiSS/KiSS1R system indicates that it may regulate several different physiological activities, many of them still unknown and/or poorly understood. The expression of KiSS and KiSS1R were firstly described in the ovary of rats during development and across the sexual cycle^19,20^. Subsequently, Shahed *et al*.^21^ found in the Siberian hamster an enhanced ovarian KiSS immunopositivity during the ovulatory transition. Few other studies have reported the expression of KiSS in various ovarian compartments, indicating additional ovarian functions of locally synthesized kisspeptins^2,8,20,22^.

Previous studies described the presence of both KiSS and KiSS1R in CL of bitches, rats, hamsters, and chickens, whereas in cow and human CL only KiSS was detected^18^. Our immunohistochemical results demonstrated that in rabbit luteal cells KiSS and KiSS1R were differentially expressed, depending on the stage of pseudopregnancy: indeed, KiSS decreased from early- to late-luteal stages, whereas KiSS1R diminished from early- to mid-luteal stage and was not detectable in late stage.

*KiSS* and *KiSS1R* gene expression has been found in the vascular system of various species^2,23^. In the present study, positive immune-reactive signals for KiSS were localized at nuclear and cytoplasm level of endothelial, and blood vessel smooth muscular cells at all stages of pseudopregnancy, whereas KiSS1R was not evidenced in blood vessel walls. The presence of KiSS in these cells is difficult to explain, even if it indirectly confirms the wide utilisation of this chemical messenger in physiological processes.

Kisspeptin directly stimulates progesterone secretion by chicken granulosa cells^24^ and rat luteal cells^9^. Progesterone is synthesised by several enzymes, including steroidogenic acute regulatory protein (StAR), cholesterol side-chain cleavage (CYP11A), cytochrome P450 side-chain cleavage (P450scc), and 3β-hydroxysteroid dehydrogenase (3β-HSD) enzymes^25^. In chicken granulosa cells, treatment *in vitro* with kisspeptin-10 upregulated the mRNA levels of all these enzymes^24^. In rat luteal cells cultured *in vitro*, kisspeptin did not affect 3β-HSD mRNA transcript, while kisspeptin and hCG together upregulated its gene expression^9^. Kisspeptin upregulated the transcripts for StAR and CYP11A, a stimulatory effect that was enhanced by co-treatment with hCG^9^. Similarly, hCG upregulated the transcript for kisspeptin in rat granulosa cells, and the hCG-mediated progesterone synthesis was blocked by the kisspeptin antagonist P234^9^. Together, these finding suggest that ovarian kisspeptin has central part in regulating progesterone production. Our present results suggest that KiSS upregulates progesterone synthesis by rabbit CL. Indeed, the *in vitro* results indicated that KiSS increased progesterone production only by CL obtained at days 4 and 9 of pseudopregnancy; by converse, KiSS had no stimulatory effect on day 13 CL, when KiSS1R is not present. This KiSS function was also supported by the results of the co-incubation with the KiSS antagonist, that blocked the progesterone increase induced by KiSS.

Prostaglandins have a pivotal role in the regulation of luteal function and lifespan: while PGF2α is the main luteolysing factor, PGE2 is the major luteoprotective factor^26–28^. Previously, using the rabbit model, we showed that these prostaglandins regulated progesterone release differently, depending on luteal stages^14,29^. PGE2 enhanced the synthesis of progesterone by the early CL, but had no effect in mid and late CL; PGF2α caused luteolysis in day 9 and 13 CL, but was ineffective on early CL. This dual mechanism prevents luteolysis to occur until day 6 of pseudopregnancy, when CL become partially responsive to PGF2α and develop luteolytic competence^16,29^. Other mammalian species such as cows^30^, pigs^31^, mares^32^, and monkeys^33^ acquire luteolytic competence at different days of the estrous cycle. In the present investigation, we found that KiSS diminished luteal PGF2α synthesis at early- and mid-luteal stages of pseudopregnancy, but increased that of PGE2 at the same two stages. The decrease during the CL life of the KiSS and KiSS1R protein expression, the latter completely absent in the late CL, suggests that the KiSS/KiSS1R system has an active physiological function in protecting the CL from luteolysis.

The conversion of arachidonic acid into PGH2 operated by PTGS1 or PTGS2 represents a critical step in synthesis of PG^34,35^. PGH2 is then transformed into four active PGs (PGE2, PGF2α, PGD2 and PGI2) via specific PG synthases^36^. However, the biosynthesis of PGF2α is peculiar because it derives from three different pathways catalysed by corresponding ketoreductases using PGH2, PGD2 or PGE2 as substrates^37^. In one of our previous study, we described an auto-amplification mechanism of luteal-derived PGF2α that induces luteolysis^16^. Depending on the stage of pseudopregnancy, PGF2α activates PGE2-9-ketoreductase and PTGS2, which converts arachidonic acid to PGH2 that is transformed into PGF2α and PGE2; this latter is then converted into PGF2α by the activated PGE2-9-ketoreductase. This enzyme has also a 20α-HSD activity^16^, in fact converts progesterone into 20α-OH-progesterone and thus contributes to the decrease of progesterone induced by PGF2α. In our investigation, KiSS did not affect PTGS1, whereas it decreased PTGS2 at mid-luteal stage and this effect was nullified by the addition of its antagonist. The KiSS antagonist showed an opposite effect on PG synthesis and counteracted those of KiSS agonist when co-incubated together.

## Conclusions

The present data evidence a novel peripheral reproductive role for the KiSS/KiSS1R system in the control of rabbit CL function exerted locally, outside the well described central hypothalamic role. In particular, KiSS plays a luteotropic role and increases luteal progesterone production likely via autocrine and/or paracrine mechanisms that simultaneously induce PGF2α decrease and PGE2 increase due to the block of the PTGS2 activity. The production of KiSS in the absence of KiSS1R expression in the late CL suggests that the KiSS/KiSS1R system activity is mainly regulated by gene and/or protein expression of the receptor.

It is well established that a variety of factors, mediating external and internal conditions, such as melatonin (environmental cues), gonadal steroids (reproductive status), and leptin (metabolic status), regulate KiSS expression at hypothalamic level^2,38^. An intriguing idea is that all or some of these factors could affect the KiSS/KiSS1R system at peripheral level also. Even though the present data provides a novel insight on physiological mechanisms regulating rabbit CL lifespan, further studies are required to better understand the fine tuning of the rabbit ovarian KiSS/KiSS1R system.

## Supplementary information

Supplementary information

## Data Availability

On reasonable request, data concerning this study can be obtained from the corresponding author.
