# Supplementary material for: Kisspeptin/kisspeptin receptor system in pseudopregnant rabbit corpora lutea: presence and function
Source: Sci Rep. 2019 Mar 25;9:5044. doi: 10.1038/s41598-019-41623-1 (PMC6433948; doi:10.1038/s41598-019-41623-1)
Supplement: Supplementary file 1 — Supplementary information [file 41598_2019_41623_MOESM1_ESM.pdf]

Kisspeptin/kisspeptin receptor system in pseudopregnant rabbit corpora lutea: presence and function.

Margherita Maranesi[1], Linda Petrucci[1], Leonardo Leonardi[1], Antonello Bufalari[1], Francesco Parillo[2,3], Cristiano Boiti[1], Massimo Zerani[1,2,]

[1]Dipartimento di Medicina veterinaria, Università di Perugia, via San Costanzo 4, Perugia IT 06126, Italy
[2]Scuola di Bioscienze e Medicina veterinaria, Università di Camerino, via Circonvallazione 93, Matelica, IT 62024, Italy
[3]Deceased

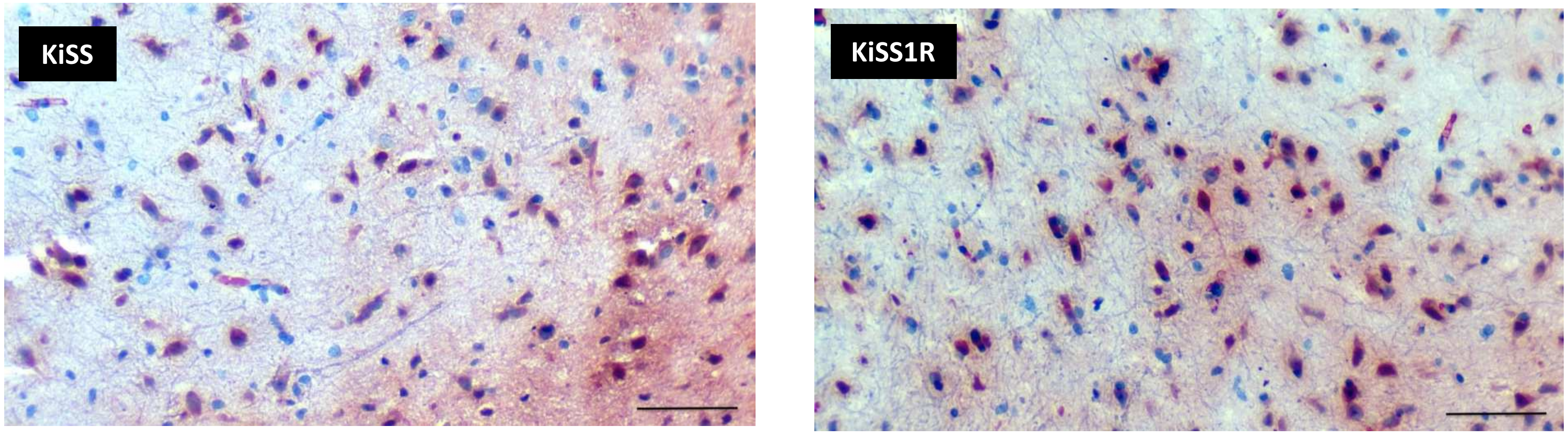

Fig. S1. Note immunosignals for KiSS (left panel) and KiSS1R (right panel) in neurons present near the III ventricle. Bars = 20 µm.

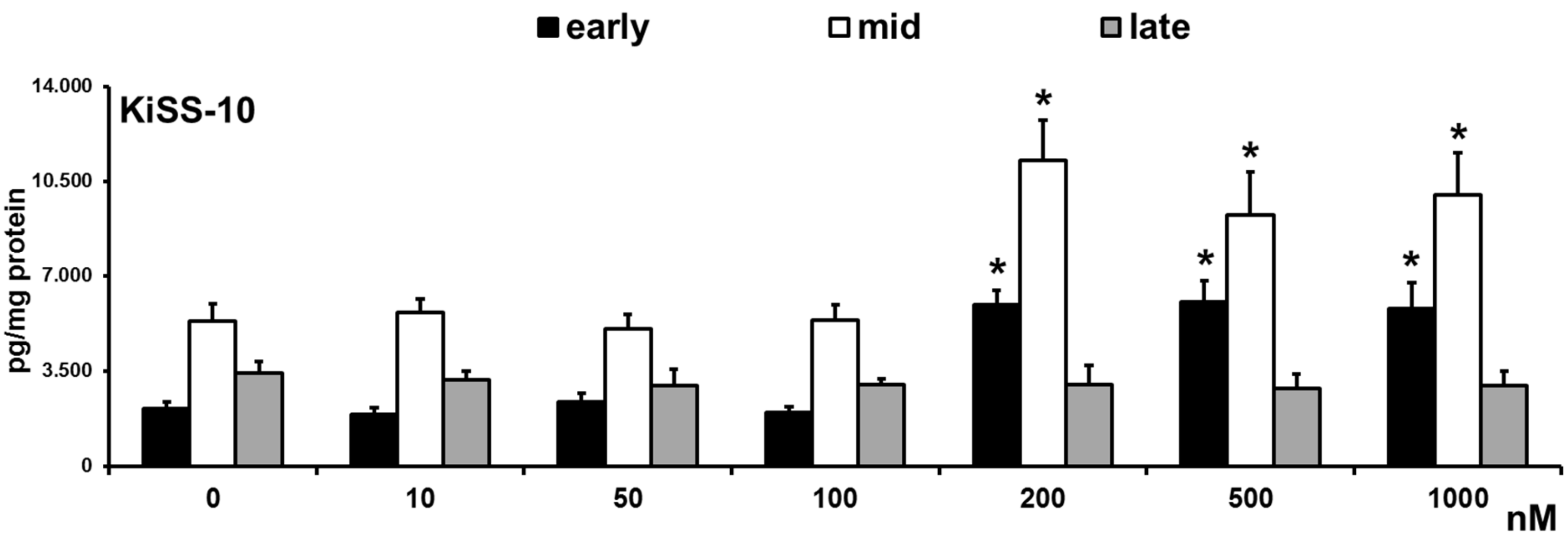

Fig. S2. In vitro effects of increasing concentrations of KiSS agonist (KiSS-10) and antagonist (KiSS-234) on progesterone release by pseudopregnant rabbit CL collected at early, mid, and late luteal stages. Values are the means ± SD of five replicates (one-way ANOVA). Asterisks indicate a significantly different value (P<0.01) versus control (0 nM).
